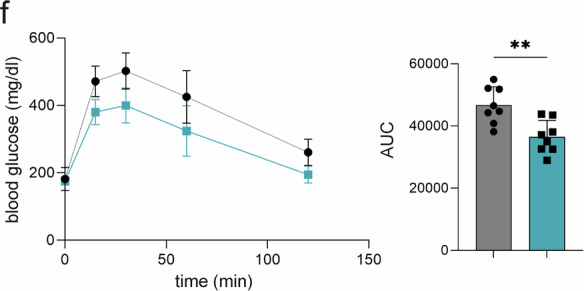# Author Correction: Regulatory T cells in the mouse hypothalamus control immune activation and ameliorate metabolic impairments in high-calorie environments

**DOI:** 10.1038/s41467-026-73173-2

**Published:** 2026-05-18

**Authors:** Maike Becker, Stefanie Kälin, Anne H. Neubig, Michael Lauber, Daria Opaleva, Hannah Hipp, Victoria K. Salb, Verena B. Ott, Beata Legutko, Roland E. Kälin, Markus Hippich, Martin G. Scherm, Lucas F. R. Nascimento, Isabelle Serr, Fabian Hosp, Alexei Nikolaev, Alma Mohebiany, Martin Krueger, Bianca Flachmeyer, Michael W. Pfaffl, Bettina Haase, Chun-Xia Yi, Sarah Dietzen, Tobias Bopp, Stephen C. Woods, Ari Waisman, Benno Weigmann, Matthias Mann, Matthias H. Tschöp, Carolin Daniel

**Affiliations:** 1 Research Unit Type 1 Diabetes Immunology, Helmholtz Diabetes Center at Helmholtz Munich, Munich, Germany; 2 German Center for Diabetes Research (DZD), Munich, Germany; 3 Institute for Diabetes and Obesity, Helmholtz Diabetes Center at Helmholtz Munich and Division of Metabolic Diseases, Technische Universität München, Munich, Germany; 4 Department of Neurosurgery, Medical Faculty, Johannes Kepler University Linz, Linz, Austria; 5 Clinical Research Institute for Neurosciences, Johannes Kepler University Linz and Kepler University Hospital, Linz, Austria; 6 Neurosurgical Research, Department of Neurosurgery, University Hospital, Ludwig-Maximilians-University Munich, Munich, Germany; 7 Institute for Diabetes Research, Helmholtz Diabetes Center at Helmholtz Munich, 80939 Munich, and Klinikum rechts der Isar, Technische Universität München, Munich, Germany; 8 Department of Proteomics and Signal Transduction, Max Planck Institute of Biochemistry, Martinsried, Germany; 9 Institute for Molecular Medicine, Universitätsmedizin der Johannes-Gutenberg-Universität, Mainz, Germany; 10 Institute for Anatomy, Leipzig University, Leipzig, Germany; 11 Animal Physiology and Immunology, Technische Universität München, Freising-Weihenstephan, Germany; 12 Genomics Core Facility, EMBL European Molecular Biology Laboratory, Heidelberg, Germany; 13 Department of Endocrinology and Metabolism, Amsterdam University Medical Center, location AMC, University of Amsterdam, Amsterdam, The Netherlands; 14 Institute of Immunology, Johannes Gutenberg University Mainz, Mainz, Germany; 15 Metabolic Diseases Institute, Department of Psychiatry and Behavioral Neuroscience, University of Cincinnati, Cincinnati, OH USA; 16 Department of Medicine 1, University of Erlangen-Nuremberg, Kussmaul Campus for Medical Research, Erlangen, Germany; 17 Division of Clinical Pharmacology, Department of Medicine IV, Ludwig-Maximilians-Universität München, Munich, Germany

**Keywords:** Immune tolerance, Neuroimmunology, Regulatory T cells, Type 2 diabetes

Correction to: *Nature Communications* 10.1038/s41467-025-57918-z, published online 20 March 2025

In the version of the article initially published, the bar chart in Fig. 7f was incorrect and has now been replaced in the HTML and PDF versions of the article, as seen in Fig. 1 below.

Original Fig. 7f:

**Fig. 1:**
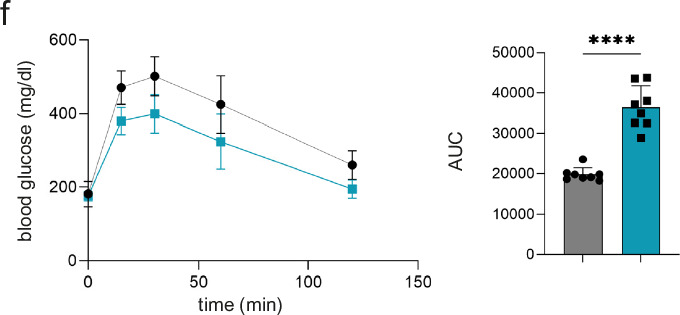
Original and corrected Fig. 7f.dd

Corrected Fig. 7f:

**Figure:**