# Supplementary material for: Regulatory T cells in the mouse hypothalamus control immune activation and ameliorate metabolic impairments in high-calorie environments
Source: Nat Commun. 2025 Mar 20;16:2744. doi: 10.1038/s41467-025-57918-z (PMC11926360; doi:10.1038/s41467-025-57918-z)
Supplement: Supplementary file 1 — Supplementary Information [file 41467_2025_57918_MOESM1_ESM.pdf]

Supplementary Information

Contains 10 Supplementary Figures

and

3 Supplementary Tables.

Regulatory T-cells in the murine hypothalamus control immune activation and improve metabolic impairments upon high-calorie environments

Authors

Maike Becker[1,2,], Stefanie Kälin[3,2,], Anne H. Neubig[1,2], Michael Lauber[1,2], Daria Opaleva[1,2], Hannah Hipp[1,2], Victoria K. Salb[1,2], Verena B. Ott[3,2], Beata Legutko[3], Roland E. Kälin[4,5], Markus Hippich[6,2], Martin G. Scherm[1,2], Lucas F. R. Nascimento[1,2], Isabelle Serr[1,2], Fabian Hosp[7], Alexei Nikolaev[8], Alma Mohebiany[8], Martin Krueger[9], Bianca Flachmeyer[9], Michael Pfaffl[10], Bettina Haase[11], Chun-Xia Yi[12], Sarah Dietzen[13], Tobias Bopp[13], Stephen C. Woods[14], Ari Waisman[8], Benno Weigmann[15], Matthias Mann[7], Matthias H. Tschöp[2,3,*] and Carolin Daniel[1,2,16,*]

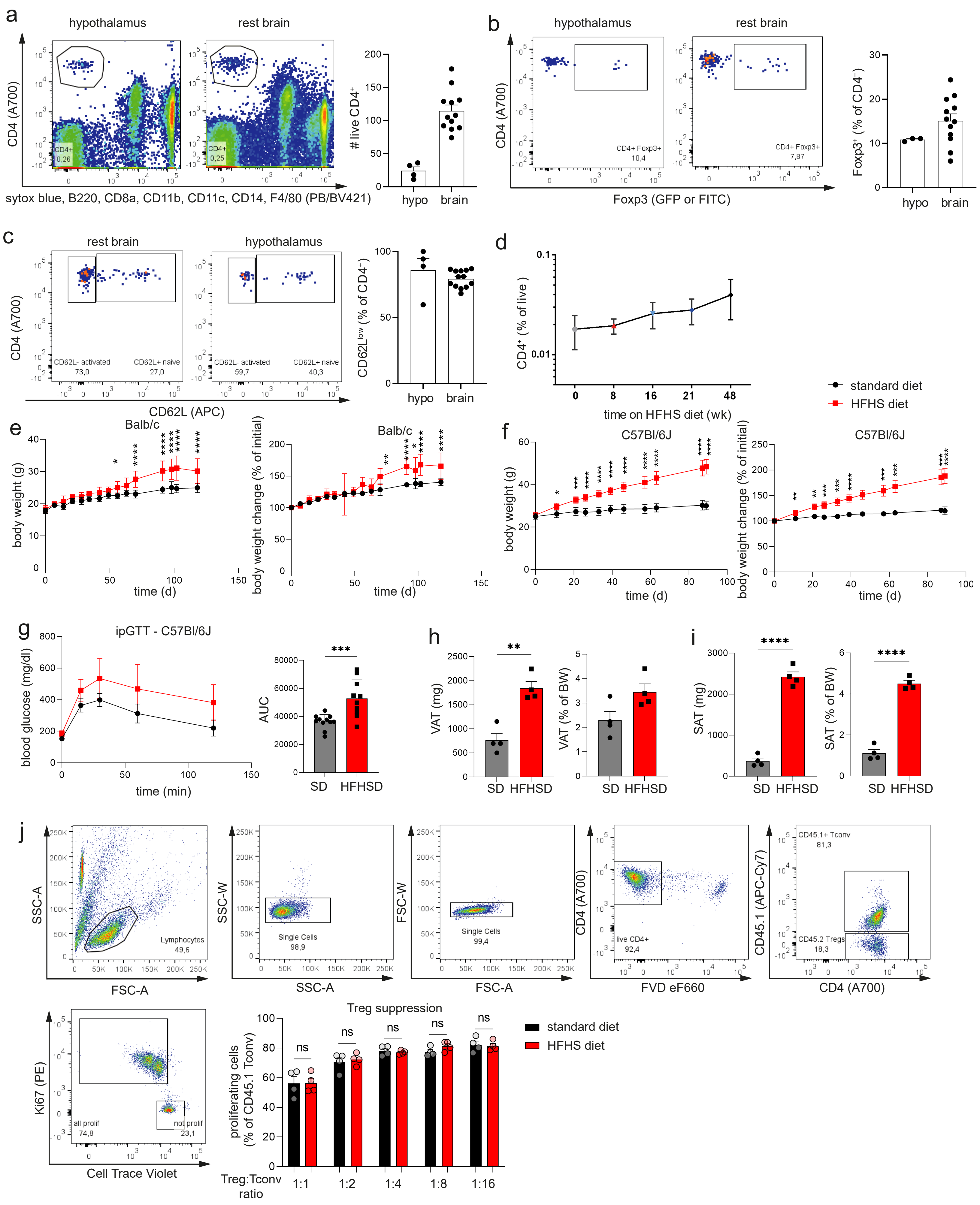
a
hypothalamus
rest brain
CD4 (A700)
CD4+ 0,26
CD4+ 0,25
sytox blue, B220, CD8a, CD11b, CD11c, CD14, F4/80 (PB/BV421)
live CD4+
hypo
brain
b
hypothalamus
rest brain
CD4 (A700)
CD4+ Foxp3+ 10,4
CD4+ Foxp3+ 7,87
Foxp3 (GFP or FITC)
Foxp3+ (% of CD4+)
hypo
brain
c
rest brain
hypothalamus
CD4 (A700)
CD62L- activated 73,0
CD62L+ naive 27,0
CD62L- activated 59,7
CD62L+ naive 40,3
CD62L (APC)
CD62L low (% of CD4+)
hypo
brain
d
CD4+ (% of live)
time on HFHS diet (wk)
standard diet
HFHS diet
e
Balb/c
body weight (g)
time (d)
body weight change (% of initial)
f
C57Bl/6J
body weight (g)
time (d)
body weight change (% of initial)
g
ipGTT - C57Bl/6J
blood glucose (mg/dl)
time (min)
AUC
SD
HFHSD
h
VAT (mg)
VAT (% of BW)
SD
HFHSD
i
SAT (mg)
SAT (% of BW)
SD
HFHSD
j
SSC-A
FSC-A
Lymphocytes 49,6
SSC-W
SSC-A
Single Cells 98,9
FSC-W
FSC-A
Single Cells 99,4
CD4 (A700)
FVD eF660
live CD4+ 92,4
CD45.1 (APC-Cy7)
CD4 (A700)
CD45.1+ Tconv 81,3
CD45.2 Tregs 18,3
Ki67 (PE)
Cell Trace Violet
all prolif 74,8
not prolif 23,1
Treg suppression
proliferating cells (% of CD45.1 Tconv)
Treg:Tconv ratio
1:1
1:2
1:4
1:8
1:16
ns
standard diet
HFHS diet

Supplementary Figure 1: Identification of CD4$^+$T cells in brains of healthy mice. Related to Figures 1 and 2:

(**a-c**) Representative FACS plots and the corresponding quantification of (a) CD4$^+$T cells, (b) Foxp3GFP$^+$Tregs and (c) CD4$^+$CD62L^{low}T cells in murine hypothalamus vs. rest brain after transcardial perfusion using 10 U/ml heparin in 0.9% NaCl. N=3-14 biological replicates. Mean±SEM. Two-tailed student's unpaired *t*-test. *P*(a)=0.0001; *p*(b)=0.2050; *p*(c)=0.2706.

(**d**) Frequencies of CD4$^+$T cells isolated from murine hypothalamus after exposure of 8-48 weeks to a HFHS diet. Mean±SD.

(**e**-**f**) Body weight curves of mice fed the standard diet vs. HFHS diet. Mean±SD. Two-way ANOVA with Šidák post-hoc test. Exact *p*-values and N numbers are provided in the Source Data file.

(**g**) ipGTT and the area under the curve (AUC) of C57Bl/6J mice fed the standard diet vs. HFHS diet for 16 weeks. Mean±SD and n=10-11 biological replicates per group. Two-tailed student's unpaired *t*-test, *p*=0.0010.

(**h-i**) Visceral adipose tissue (VAT, h) and subcutaneous adipose tissue (SAT, i) mass at the end of the study (of g). Mean±SEM and n=4 biological replicates. Two-tailed student's unpaired *t*-test, *p(*VAT, mg*)*=0.0014; *p(*VAT, %*)*=0.0561; *p(*SAT, mg*)*<0.0001; *p(*SAT, %*)*<0.0001.

(**j**) Representative FACS plots and the corresponding quantification of an *in vitro* Treg suppression assay using naïve CD45.1 Tconv cells that were suppressed by Tregs from mice that were fed with the standard diet or the HFHS diet as in (a). Treg:Tconv ratio was titrated from 1:1 to 1:16. n=4 biological replicates per group. Two-tailed student's unpaired *t*-test per condition with *p*(1:1)=0.9895; *p*(1:2)=0.6641; *p*(1:4)=0.5605; *p*(1:8)=0.1410; *p*(1:2)=0.7955.

Source data are provided as a Source Data file. *=*p*<0.05; **=*p*<0.01, ***=*p*<0.001, ***=*p*<0.0001.

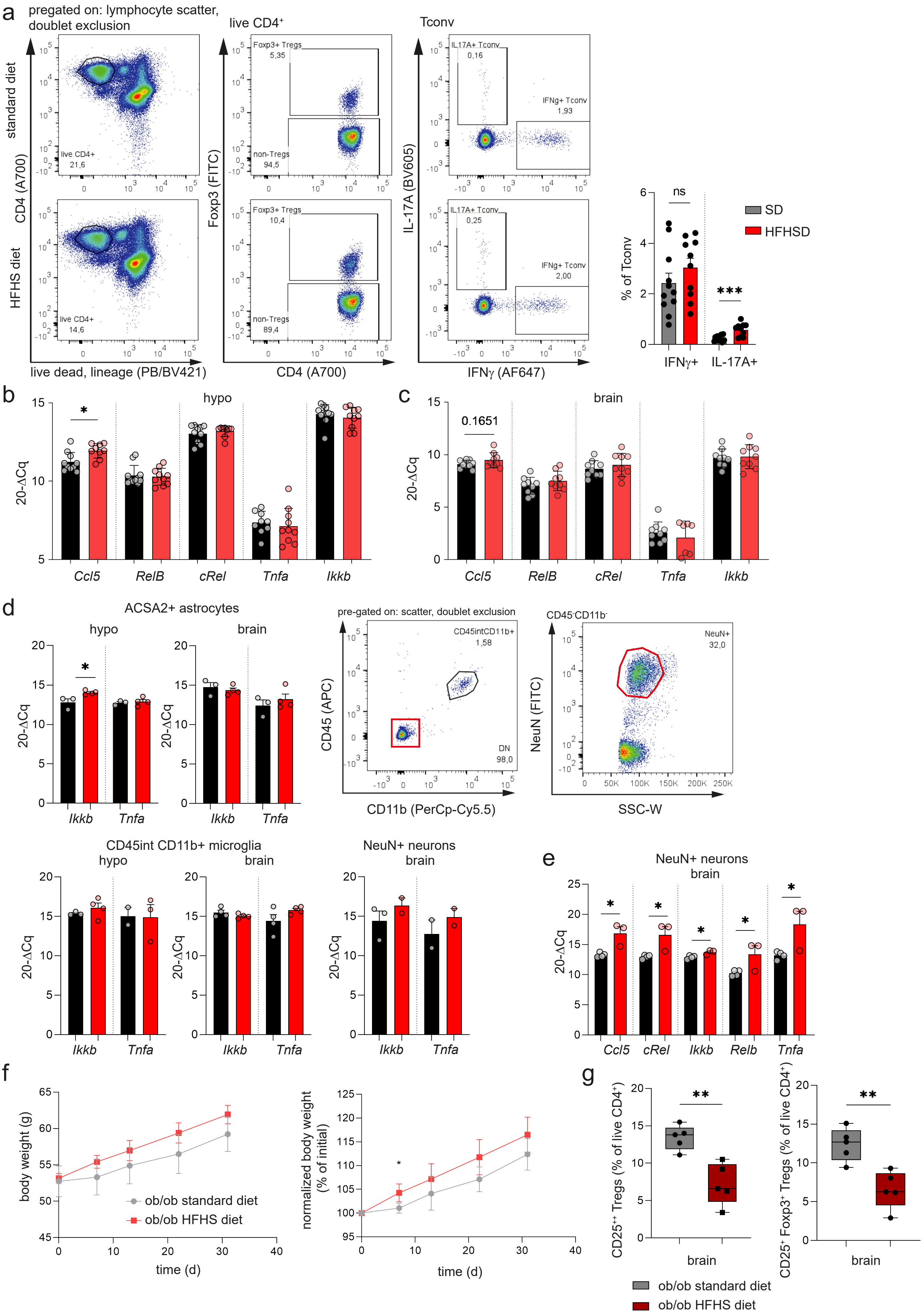
a
pregated on: lymphocyte scatter, doublet exclusion
live CD4+
Tconv
standard diet
HFHS diet
CD4 (A700)
live CD4+ 21,6
live CD4+ 14,6
live dead, lineage (PB/BV421)
Foxp3+ Tregs 5,35
non-Tregs 94,5
Foxp3+ Tregs 10,4
non-Tregs 89,4
Foxp3 (FITC)
CD4 (A700)
IL17A+ Tconv 0,16
IFNg+ Tconv 1,93
IL17A+ Tconv 0,25
IFNg+ Tconv 2,00
IL-17A (BV605)
IFNγ (AF647)
% of Tconv
ns
***
SD
HFHSD
IFNγ+
IL-17A+
b
hypo
20-ΔCq
Ccl5
RelB
cRel
Tnfa
Ikkb
c
brain
0.1651
d
ACSA2+ astrocytes
hypo
brain
pre-gated on: scatter, doublet exclusion
CD45intCD11b+ 1,58
DN 98,0
CD45 (APC)
CD11b (PerCp-Cy5.5)
CD45-CD11b-
NeuN+ 32,0
NeuN (FITC)
SSC-W
CD45int CD11b+ microglia
NeuN+ neurons
e
NeuN+ neurons brain
Relb
f
body weight (g)
time (d)
ob/ob standard diet
ob/ob HFHS diet
normalized body weight (% of initial)
g
CD25++ Tregs (% of live CD4+)
CD25+ Foxp3+ Tregs (% of live CD4+)
brain
**

Supplementary Figure 2: Cytokine profiles of T cells and suppressive capacity of Tregs isolated from mice fed a HFHS diet. Related to Figure 2.

(**a**) Representative FACS plots and the corresponding quantification of cytokine expression in $CD4^{+}$T cells isolated from lymph nodes of wildtype C57Bl/6J mice fed a standard diet vs HFHS diet. n=10-11 biological replicates per group. Two-tailed Student's unpaired *t*-test with *p*(IFNg)=0.2780; *p*(IL17A)=0.0006.

(**b-c**) Gene expression analyses of (b) hypothalamic and (c) rest brains of standard diet vs HFHS diet-fed wildtype C57Bl/6J mice. Gene expression was normalized to *Histone*. Mean±SEM. N=9-11 biological replicates per group. Two-tailed Student's unpaired *t*-test. Hypothalamus: *p*(*Ccl5*)=0.0115*;* *p*(*Relb*)=0.7463; *p*(*cRel*)=0.4826; *p*(*Tnfa*)=0.6135; *p*(*Ikkb*)=0.403. Brain: *p*(*Ccl5*)=0.1651*;* *p*(*Relb*)=0.2800; *p*(*cRel*)=0.4284; *p*(*Tnfa*)=0.9020; *p*(*Ikkb*)=0.7943.

(**d**) Gene expression analyses of sorted cell populations. $ACSA2^{+}$ astrocytes were MACS-sorted. For sorting plots for microglia ($CD45^{int}CD11b^{+}$) and Neurons ($CD45^{-}CD11b^{-}NeuN^{+}$) see representative FACS plots. Sorted cell populations were from either standard diet- or 16 wk HFHS diet fed wildtype C57Bl/6J mice. Gene expression was normalized to *Histone H3* and plotted as 20-Delta Cq. N=3-4 biological replicates per group. Mean±SEM. Two-tailed Student's unpaired *t*-test. Hypothalamus: *p*(*Ikkb*)=0.0278; *p*(*Tnfa*)=0.6712; Brain: *p*(*Ikkb*)=0.5421; *p*(*Ikkb*)=0.4495.

(**e**) FACS-sorted NeuN+ neurons from rest brains of Balb/c mice fed a standard diet vs. a HFHS diet for 16 wk. Gene expression was normalized to *Histone H3* and plotted as 20-Delta Cq. N=3-4 biological replicates per group. Mean±SEM. Two-tailed Student's unpaired *t*-test with *p*(*Ccl5*)=0.0124; *p*(*cRel*)=0.0252; *p*(*Ikkb*)=0.0178; *p*(*Relb*)=0.0488; p(Tnfa)=0.0403.

(**f**) Body weight curves of ob/ob mice fed a standard diet or HFHS diet. n=5 biological replicates per group. Mean±SD.

(**g**) Treg frequencies in brains of ob/ob mice of (E). n=5 biological replicates per group. Depicted are box-and-whisker plots (min to max with all data points).Two-tailed Student's unpaired *t*-test. *p*($CD25^{+++}$)=0.0025; *p*(*Foxp3*+)=0.0037.

Source data are provided as a Source Data file. *=*p*<0.05; **=*p*<0.01.

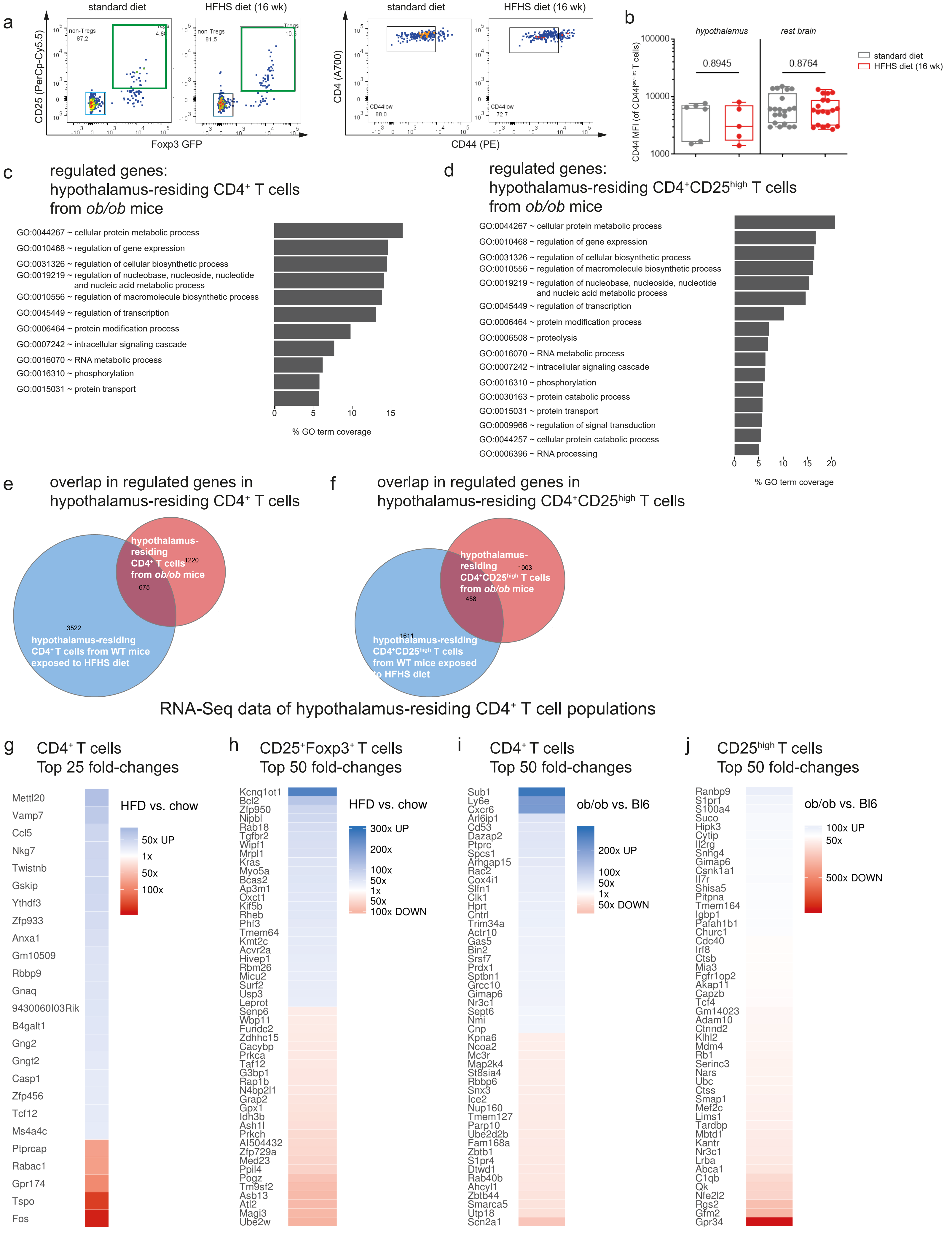

a
standard diet
HFHS diet (16 wk)
CD25 (PerCp-Cy5.5)
Foxp3 GFP
non-Tregs
87,2
Tregs
4,68
non-Tregs
81,5
Tregs
10,5
CD4 (A700)
CD44 (PE)
CD44low
88,0
CD44low
72,7
b
hypothalamus
rest brain
standard diet
HFHS diet (16 wk)
CD44 MFI (of CD44low+int T cells)
0.8945
0.8764
100000
10000
1000
c
regulated genes:
hypothalamus-residing CD4+ T cells
from ob/ob mice
GO:0044267 ~ cellular protein metabolic process
GO:0010468 ~ regulation of gene expression
GO:0031326 ~ regulation of cellular biosynthetic process
GO:0019219 ~ regulation of nucleobase, nucleoside, nucleotide and nucleic acid metabolic process
GO:0010556 ~ regulation of macromolecule biosynthetic process
GO:0045449 ~ regulation of transcription
GO:0006464 ~ protein modification process
GO:0007242 ~ intracellular signaling cascade
GO:0016070 ~ RNA metabolic process
GO:0016310 ~ phosphorylation
GO:0015031 ~ protein transport
% GO term coverage
d
regulated genes:
hypothalamus-residing CD4+CD25high T cells
from ob/ob mice
GO:0044267 ~ cellular protein metabolic process
GO:0010468 ~ regulation of gene expression
GO:0031326 ~ regulation of cellular biosynthetic process
GO:0010556 ~ regulation of macromolecule biosynthetic process
GO:0019219 ~ regulation of nucleobase, nucleoside, nucleotide and nucleic acid metabolic process
GO:0045449 ~ regulation of transcription
GO:0006464 ~ protein modification process
GO:0006508 ~ proteolysis
GO:0016070 ~ RNA metabolic process
GO:0007242 ~ intracellular signaling cascade
GO:0016310 ~ phosphorylation
GO:0030163 ~ protein catabolic process
GO:0015031 ~ protein transport
GO:0009966 ~ regulation of signal transduction
GO:0044257 ~ cellular protein catabolic process
GO:0006396 ~ RNA processing
% GO term coverage
e
overlap in regulated genes in
hypothalamus-residing CD4+ T cells
hypothalamus-residing CD4+ T cells from ob/ob mice
1220
675
3522
hypothalamus-residing CD4+ T cells from WT mice exposed to HFHS diet
f
overlap in regulated genes in
hypothalamus-residing CD4+CD25high T cells
hypothalamus-residing CD4+CD25high T cells from ob/ob mice
1003
458
1611
hypothalamus-residing CD4+CD25high T cells from WT mice exposed to HFHS diet
RNA-Seq data of hypothalamus-residing CD4+ T cell populations
g
CD4+ T cells
Top 25 fold-changes
HFD vs. chow
50x UP
1x
50x
100x
Mettl20
Vamp7
Ccl5
Nkg7
Twistnb
Gskip
Ythdf3
Zfp933
Anxa1
Gm10509
Rbbp9
Gnaq
9430060I03Rik
B4galt1
Gng2
Gngt2
Casp1
Zfp456
Tcf12
Ms4a4c
Ptprcap
Rabac1
Gpr174
Tspo
Fos
h
CD25+Foxp3+ T cells
Top 50 fold-changes
HFD vs. chow
300x UP
200x
100x
50x
1x
50x
100x DOWN
Kcnq1ot1
Bcl2
Zfp950
Nipbl
Rab18
Tgfbr2
Wipf1
Mrpl1
Kras
Myo5a
Bcas2
Ap3m1
Oxct1
Kif5b
Rheb
Phf3
Tmem64
Kmt2c
Acvr2a
Hivep1
Rbm26
Micu2
Surf2
Usp3
Leprot
Senp6
Wbp11
Fundc2
Zdhhc15
Cacybp
Prkca
Taf12
G3bp1
Rap1b
N4bp2l1
Grap2
Gpx1
Idh3b
Ash1l
Prkch
AI504432
Zfp729a
Med23
Ppil4
Pogz
Tm9sf2
Asb13
Atl2
Magi3
Ube2w
i
CD4+ T cells
Top 50 fold-changes
ob/ob vs. Bl6
200x UP
100x
50x
1x
50x DOWN
Sub1
Ly6e
Cxcr6
Arl6ip1
Cd53
Dazap2
Ptprc
Spcs1
Arhgap15
Rac2
Cox4i1
Slfn1
Clk1
Hprt
Cntrl
Trim34a
Actr10
Gas5
Bin2
Srsf7
Prdx1
Sptbn1
Grcc10
Gimap6
Nr3c1
Sept6
Nmi
Cnp
Kpna6
Ncoa2
Mc3r
Map2k4
St8sia4
Rbbp6
Snx3
Ice2
Nup160
Tmem127
Parp10
Ube2d2b
Fam168a
Zbtb1
S1pr4
Dtwd1
Rab40b
Ahcyl1
Zbtb44
Smarca5
Utp18
Scn2a1
j
CD25high T cells
Top 50 fold-changes
ob/ob vs. Bl6
100x UP
50x
500x DOWN
Ranbp9
S1pr1
S100a4
Suco
Hipk3
Cytip
Il2rg
Snhg4
Gimap6
Csnk1a1
Il7r
Shisa5
Pitpna
Tmem164
Igbp1
Pafah1b1
Churc1
Cdc40
Irf8
Ctsb
Mia3
Fgfr1op2
Akap11
Capzb
Tcf4
Gm14023
Adam10
Ctnnd2
Klhl2
Mdm4
Rb1
Serinc3
Nars
Ubc
Ctss
Smap1
Mef2c
Lims1
Tardbp
Mbtd1
Kantr
Nr3c1
Lrba
Abca1
C1qb
Qk
Nfe2l2
Rgs2
Gfm2
Gpr34

Supplementary Figure 3: Transcriptome analyses of hypothalamic $CD4^{+}$T cells. Related to Figure 3:

(**a**) Representative sorting scheme for T cells used for RNA-Seq showing $CD4^{+}CD25^{-}$ Foxp3 $GFP^{-}CD44^{low+int}$ and $CD4^{+}CD25^{hi}$Foxp3 GFP^{+} cells. Cells were pre-gated based on lymphocyte scatter, doublet exclusion, and live $CD4^{+}$ T cells.

(**b**) analyses of the mean fluorescence intensity (MFI) of CD44 abundance on $CD4^{+}CD25^{-}$ Foxp3 $GFP^{-}CD44^{low+int}$ T cells as in (a) to demonstrate the absence of a difference in maturational status of sorted input cell populations used for sequencing experiments. N=5-21 biological replicates. Two-tailed Student's unpaired *t*-test with $p(Hypo)=0.5888$; $p(brain)=0.6646$.

(**c-d**) DESeq2 normalized read counts regulated more than 2.5-fold (*ob/ob* vs. C57Bl/6J) were functionally annotated to Gene Ontology Biological Processes (GOBP) level 5 using DAVID Bioinformatics Resources 6.7. Terms are depicted as percentage GO term gene coverage for (**c**) hypothalamic $CD4^{+}$T cells and (**d**) hypothalamic $CD4^{+}CD25^{high}Foxp3^{+}$T cells.

(**e-f**) Venn-diagram of regulated genes of (**e**) hypothalamic $CD4^{+}$T cells and (**f**) $CD4^{+}CD25^{high}Foxp3^{+}$T cells from *ob/ob* mice and C57Bl/6J mice fed HFHS diet.

(**g-j**) Top fold-changes of DESeq2 normalized read counts of (g) $CD4^{+}$ and (h) $CD4^{+}CD25^{+}Foxp3GFP^{+}$ T cell populations from HFHS diet- vs. SD-fed Foxp3GFP reporter mice, as well as of (i) $CD4^{+}$ T cells and (j) $CD4^{+}CD25^{hi}$ T cells from *ob/ob* vs. C57Bl/6J mice. A cutoff of 30 reads was applied.

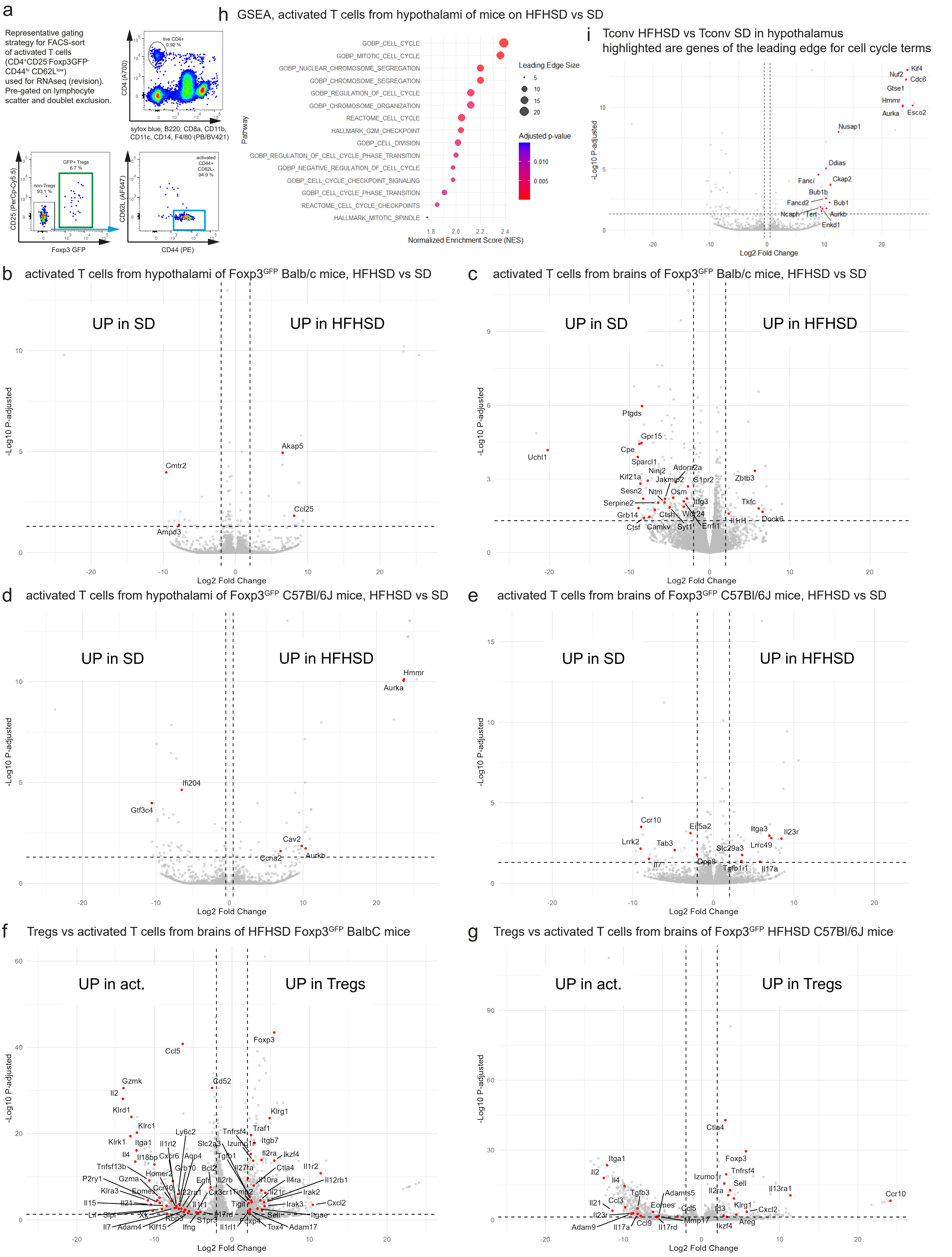

a
Representative gating strategy for FACS-sort of activated T cells (CD4+CD25-Foxp3GFP- CD44hi CD62Llow) used for RNAseq (revision). Pre-gated on lymphocyte scatter and doublet exclusion.
live CD4+ 0.92 %
CD4 (A700)
sytox blue, B220, CD8a, CD11b, CD11c, CD14, F4/80 (PB/BV421)
GFP+ Tregs 6.7 %
non-Tregs 93.1 %
CD25 (PerCp-Cy5.5)
Foxp3 GFP
activated CD44+ CD62L- 94.9 %
CD62L (AF647)
CD44 (PE)
h
GSEA, activated T cells from hypothalami of mice on HFHSD vs SD
GOBP_CELL_CYCLE
GOBP_MITOTIC_CELL_CYCLE
GOBP_NUCLEAR_CHROMOSOME_SEGREGATION
GOBP_CHROMOSOME_SEGREGATION
GOBP_REGULATION_OF_CELL_CYCLE
GOBP_CHROMOSOME_ORGANIZATION
REACTOME_CELL_CYCLE
HALLMARK_G2M_CHECKPOINT
GOBP_CELL_DIVISION
GOBP_REGULATION_OF_CELL_CYCLE_PHASE_TRANSITION
GOBP_NEGATIVE_REGULATION_OF_CELL_CYCLE
GOBP_CELL_CYCLE_CHECKPOINT_SIGNALING
GOBP_CELL_CYCLE_PHASE_TRANSITION
REACTOME_CELL_CYCLE_CHECKPOINTS
HALLMARK_MITOTIC_SPINDLE
Pathway
Normalized Enrichment Score (NES)
Leading Edge Size
Adjusted p-value
i
Tconv HFHSD vs Tconv SD in hypothalamus
highlighted are genes of the leading edge for cell cycle terms
Kif4, Nuf2, Cdc6, Gtse1, Hmmr, Aurka, Esco2, Nusap1, Ddias, Fanci, Ckap2, Bub1b, Fancd2, Bub1, Ncaph, Tert, Aurkb, Enkd1
-Log10 P-adjusted
Log2 Fold Change
b
activated T cells from hypothalami of Foxp3GFP Balb/c mice, HFHSD vs SD
UP in SD
UP in HFHSD
Akap5, Cmtr2, Ccl25, Ampd3
-Log10 P-adjusted
Log2 Fold Change
c
activated T cells from brains of Foxp3GFP Balb/c mice, HFHSD vs SD
UP in SD
UP in HFHSD
Ptgds, Gpr15, Uchl1, Cpe, Sparcl1, Ninj2, Adora2a, Kif21a, Jakmip2, S1pr2, Zbtb3, Sesn2, Ntm, Osm, Tkfc, Serpine2, Itfg3, Grb14, Ctsh, Wdr24, Il1rl1, Dock6, Ctsf, Camkv, Syt1, Errfi1
-Log10 P-adjusted
Log2 Fold Change
d
activated T cells from hypothalami of Foxp3GFP C57Bl/6J mice, HFHSD vs SD
UP in SD
UP in HFHSD
Hmmr, Aurka, Ifi204, Gtf3c4, Cav2, Ccna2, Aurkb
-Log10 P-adjusted
Log2 Fold Change
e
activated T cells from brains of Foxp3GFP C57Bl/6J mice, HFHSD vs SD
UP in SD
UP in HFHSD
Ccr10, Eif5a2, Itga3, Il23r, Lrrk2, Tab3, Slc29a3, Lrrc49, Il7, Dpp8, Tgfb1i1, Il17a
-Log10 P-adjusted
Log2 Fold Change
f
Tregs vs activated T cells from brains of HFHSD Foxp3GFP BalbC mice
UP in act.
UP in Tregs
Foxp3, Ccl5, Gzmk, Cd52, Il2, Klrd1, Klrg1, Klrc1, Ly6c2, Tnfrsf4, Traf1, Klrk1, Itga1, Il1rl2, Slc2a3, Izumo1r, Itgb7, Il4, Il18bp, Cxcr6, Aqp4, Tgfb1, Il2ra, Ikzf4, Tnfsf13b, Grb10, Bcl2, Il27ra, Ctla4, Il1r2, P2ry1, Gzma, Homer2, Egfr, Il2rb, Il10ra, Il4ra, Il12rb1, Klra3, Eomes, Ccr10, Il22ra1, Cx3cr1, Tmp2, Il21r, Irak2, Il15, Il21, Il1r1, Tigit, Irak3, Cxcl2, Lif, Slpi, Xk, Il17rd, Sell, Itgae, Rbp3, S1pr3, Pdxp4, Il7, Adam4, Klf15, Ifng, Il1rl1, Tox4, Adam17
-Log10 P-adjusted
Log2 Fold Change
g
Tregs vs activated T cells from brains of Foxp3GFP HFHSD C57Bl/6J mice
UP in act.
UP in Tregs
Ctla4, Itga1, Foxp3, Tnfrsf4, Il2, Il4, Izumo1r, Sell, Il2ra, Il13ra1, Ccr10, Tgfb3, Adamts5, Il21, Ccl3, Eomes, Ccl5, Id3, Klrg1, Cxcl2, Il23r, Areg, Adam9, Il17a, Ccl9, Il17rd, Mmp17, Ikzf4
-Log10 P-adjusted
Log2 Fold Change

Supplementary Figure 4: Transcriptome analysis of hypothalamic $CD4^{+}T$ cells. Related to Figure 3:

(**a**) Representative FACS plot for activated T cells (non-Tregs) used for RNA-Seq (revision). Cells were pre-gated based on lymphocyte scatter, doublet exclusion. Activated T cells (non-Tregs) were gated as live $CD4^{+}CD25^{-}Foxp3GFP^{-}CD44^{high}CD62L^{low}$ and Tregs were gated as live $CD4^{+}CD25^{+}Foxp3GFP^{+}$. The final Treg gate is shown in green, the final activated T cell gate shown in blue. Depicted is a representative plot for cells isolated from perfused brains of $Foxp3^{GFP}$ reporter mice on 16-18 wks of a HFHSD, n=5 mice were pooled for each sequencing sample.

(**b-e**) Volcano plot of differentially expressed genes in FACS-sorted activated T cells (as shown in a), comparing T cells from (b, d) hypothalami and (c, e) brains of $Foxp3^{GFP}$ reporter mice that were on a HFHSD vs SD.

(**f-g**) Volcano plot of differentially expressed genes comparing Tregs vs activated T cells isolated from brains of mice on a HFHSD.

(**h**) Gene set enrichment analysis of activated T cells from hypothalami on mice on a HFHSD vs SD. A positive normalized enrichment score (NES) refers to gene sets upregulated in response to hypercaloric feeding.

(**i**) Volcano plot for activated T cells isolated from hypothalami of HFHS diet vs. SD mice in which genes of the leading edge (of h) on cell cycle terms are highlighted in red.

a regulated genes:
hypothalamus-residing CD4^{+} T cells
from mice exposed to HFHS diet:
terms related to metabolism

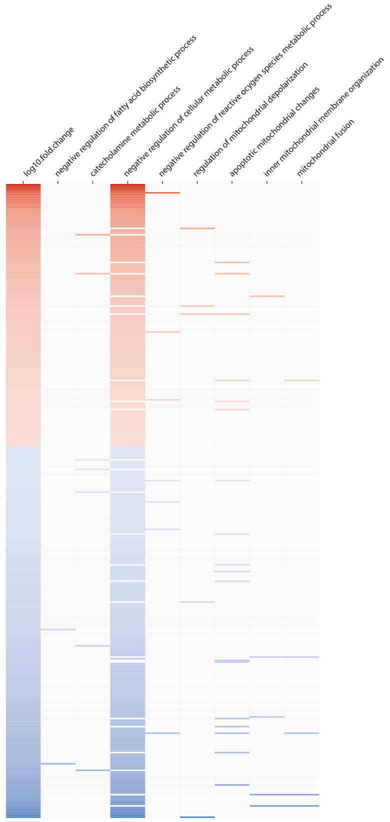

b regulated genes:
hypothalamus-residing CD4^{+}CD25^{high}Foxp3^{+} T cells
from mice exposed to HFHS diet:
terms related to metabolism

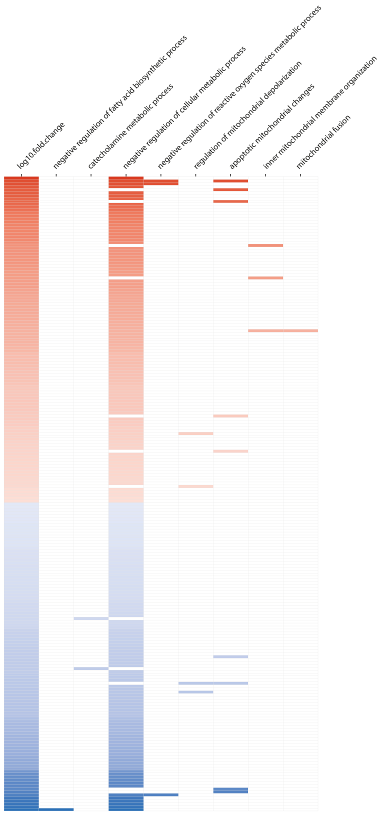

c Regulated genes in hypothalamic
CD4^{+} T cells from *ob/ob* mice:
terms related to metabolism

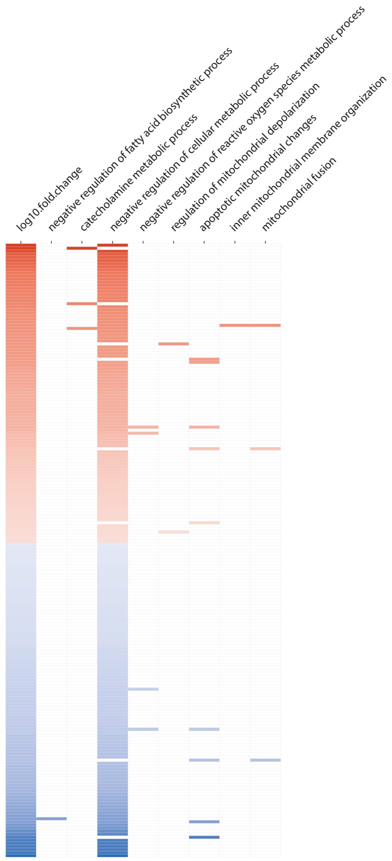

d Regulated genes in hypothalamic
CD4^{+}CD25^{high} T cells from *ob/ob* mice:
terms related to metabolism

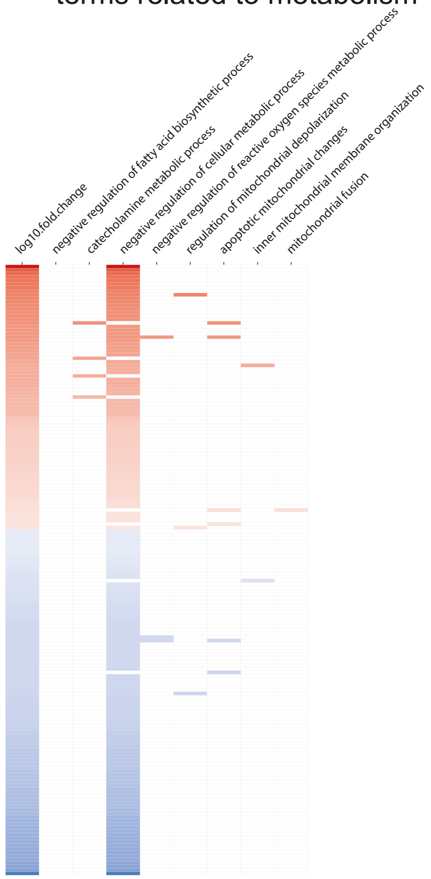

Supplementary Figure 5: Transcriptome analysis of hypothalamic CD4$^+$T cells. Related to Figure 3:

(a-b) Regulated genes (HFHS vs. standard diet) were annotated to selected metabolism-associated GOBP terms and color-coded by log_{10}-fold-change; (a) hypothalamic CD4$^+$ T cells and (b) hypothalamic CD4$^+$CD25^{high}Foxp3$^+$ T cells from mice exposed to the HFHS diet.

(c-d) Regulated genes (*ob/ob* vs. HFHS diet-fed C57Bl/6J mice) were annotated to selected metabolism-associated GOBP terms and color-coded by log_{10}-fold-change; (c) hypothalamic CD4$^+$ T cells and (d) hypothalamic CD4$^+$CD25^{high} T cells.

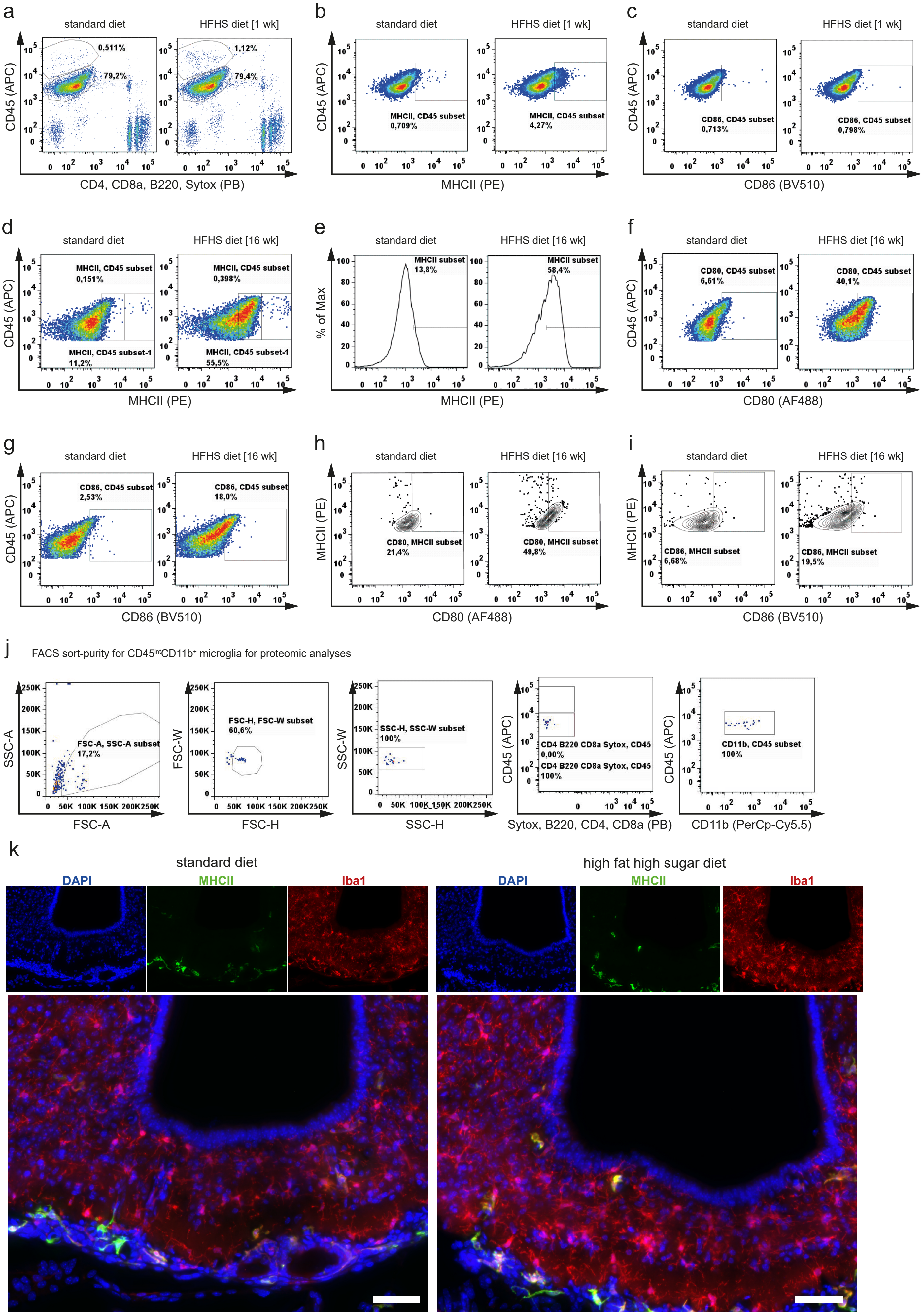
a
standard diet
HFHS diet [1 wk]
0,511%
79,2%
1,12%
79,4%
CD45 (APC)
CD4, CD8a, B220, Sytox (PB)
b
standard diet
HFHS diet [1 wk]
MHCII, CD45 subset
0,709%
MHCII, CD45 subset
4,27%
CD45 (APC)
MHCII (PE)
c
standard diet
HFHS diet [1 wk]
CD86, CD45 subset
0,713%
CD86, CD45 subset
0,798%
CD45 (APC)
CD86 (BV510)
d
standard diet
HFHS diet [16 wk]
MHCII, CD45 subset
0,151%
MHCII, CD45 subset-1
11,2%
MHCII, CD45 subset
0,398%
MHCII, CD45 subset-1
55,5%
CD45 (APC)
MHCII (PE)
e
standard diet
HFHS diet [16 wk]
MHCII subset
13,8%
MHCII subset
58,4%
% of Max
MHCII (PE)
f
standard diet
HFHS diet [16 wk]
CD80, CD45 subset
6,61%
CD80, CD45 subset
40,1%
CD45 (APC)
CD80 (AF488)
g
standard diet
HFHS diet [16 wk]
CD86, CD45 subset
2,53%
CD86, CD45 subset
18,0%
CD45 (APC)
CD86 (BV510)
h
standard diet
HFHS diet [16 wk]
CD80, MHCII subset
21,4%
CD80, MHCII subset
49,8%
MHCII (PE)
CD80 (AF488)
i
standard diet
HFHS diet [16 wk]
CD86, MHCII subset
6,68%
CD86, MHCII subset
19,5%
MHCII (PE)
CD86 (BV510)
j
FACS sort-purity for CD45intCD11b+ microglia for proteomic analyses
FSC-A, SSC-A subset
17,2%
SSC-A
FSC-A
FSC-H, FSC-W subset
60,6%
FSC-W
FSC-H
SSC-H, SSC-W subset
100%
SSC-W
SSC-H
CD4 B220 CD8a Sytox, CD45
0,00%
CD4 B220 CD8a Sytox, CD45
100%
CD45 (APC)
Sytox, B220, CD4, CD8a (PB)
CD11b, CD45 subset
100%
CD45 (APC)
CD11b (PerCp-Cy5.5)
k
standard diet
high fat high sugar diet
DAPI
MHCII
Iba1
DAPI
MHCII
Iba1

Supplementary Figure 6: Immune activation of microglia upon hyper-caloric challenge. Related to Figure 4:

(**a-i**) Antigen-presenting functions of (**a**) $CD45^{int}CD11b^{+}$ microglia upon exposure to HFHS diet (**a-c**) for 1 wk or (**d-i**) 16 wk as assessed by co-stainings of CD80, CD86 and MHCII.

(**j**) Representative FACS plot for sort-purity of $CD45^{int}CD11b^{+}$ microglia used for proteomic analyses.

(**k**) Immunofluorescence of nuclei (DAPI, blue), microglia (Iba1, red), MHCII (green) and the merged image of mice exposed to standard diet or one year of HFHS diet. The scale bar is 50 µm.

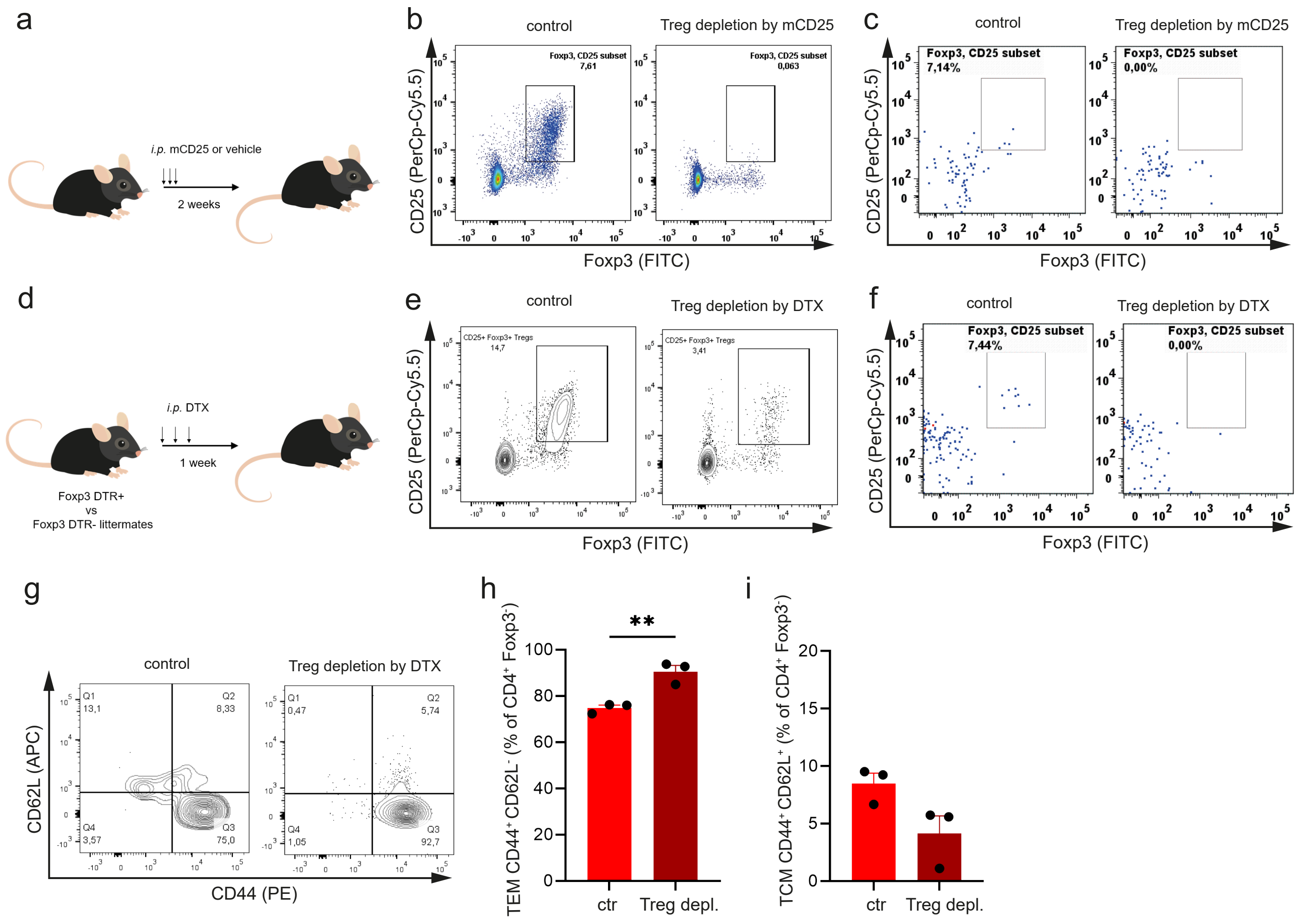

a
i.p. mCD25 or vehicle
2 weeks
b
control
Treg depletion by mCD25
Foxp3, CD25 subset
7,61
Foxp3, CD25 subset
0,063
CD25 (PerCp-Cy5.5)
Foxp3 (FITC)
c
control
Treg depletion by mCD25
Foxp3, CD25 subset
7,14%
Foxp3, CD25 subset
0,00%
CD25 (PerCp-Cy5.5)
Foxp3 (FITC)
d
i.p. DTX
1 week
Foxp3 DTR+
vs
Foxp3 DTR- littermates
e
control
Treg depletion by DTX
CD25+ Foxp3+ Tregs
14,7
CD25+ Foxp3+ Tregs
3,41
CD25 (PerCp-Cy5.5)
Foxp3 (FITC)
f
control
Treg depletion by DTX
Foxp3, CD25 subset
7,44%
Foxp3, CD25 subset
0,00%
CD25 (PerCp-Cy5.5)
Foxp3 (FITC)
g
control
Treg depletion by DTX
Q1
13,1
Q2
8,33
Q4
3,57
Q3
75,0
Q1
0,47
Q2
5,74
Q4
1,05
Q3
92,7
CD62L (APC)
CD44 (PE)
h
**
TEM CD44+ CD62L- (% of CD4+ Foxp3-)
ctr
Treg depl.
i
TCM CD44+ CD62L+ (% of CD4+ Foxp3-)
ctr
Treg depl.

Supplementary Figure 7: Two loss-of-function models of *in vivo* Treg depletion. Related to Figure 5 and 6:

(**a**) Scheme of the *in vivo* Treg depletion using *i.p*. administration of mCD25 antibodies.

(**b-c**) Representative FACS plots showing the efficacy of the Treg depletion by *i.p*. mCD25 administration in (b) inguinal LNs and (c) hypothalamus after 1 wk HFHS diet as determined by intracellular staining for Foxp3.

(**d**) Scheme of the *in vivo* Treg depletion using *i.p.* diphtheria toxin (DTX) administration in Foxp3 DTR mice.

(**e-f**) Representative FACS plots showing the efficacy of Treg depletion by *i.p.* diphtheria toxin (DTX) administration in Foxp3 DTR mice. Shown are the effects on the $CD4^+$T cell population in (e) inguinal LNs and the (f) hypothalamus after 2 wk of HFHS diet as determined by intracellular staining for Foxp3.

(**g-i**) Representative FACS plots and quantification of (h) $CD4^+Foxp3^-CD44^{hi}CD62L^{low}$ effector memory T cells (TEM) and (i) $CD4^+Foxp3^-CD44^{hi}CD62L^+$ central memory T cells (TCM) in rest brains of Foxp3 DTR mice with or without Treg depletion by *i.p.* DTX administration. N=3 biological replicates per group. Mean±SEM. Two-tailed student's unpaired *t*-test, p(TEM)=0.2592; p(TCM)=0.0706.

Source data are provided as a Source Data file. *=p<0.05; **=p<0.01.

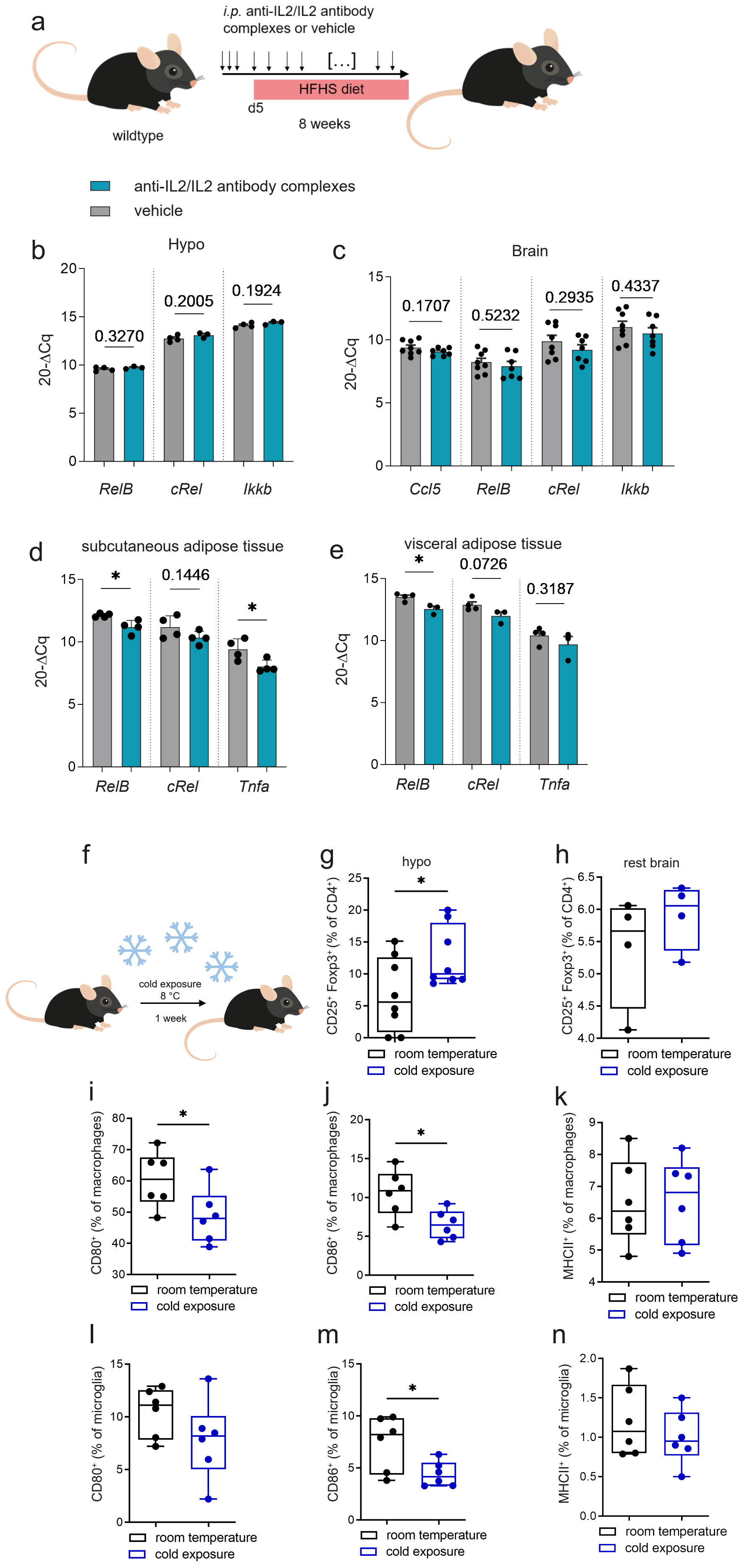
a
i.p. anti-IL2/IL2 antibody complexes or vehicle
[...]
HFHS diet
d5
8 weeks
wildtype
anti-IL2/IL2 antibody complexes
vehicle
b
Hypo
20-ΔCq
0.3270
0.2005
0.1924
RelB
cRel
Ikkb
c
Brain
20-ΔCq
0.1707
0.5232
0.2935
0.4337
Ccl5
RelB
cRel
Ikkb
d
subcutaneous adipose tissue
20-ΔCq
*
0.1446
*
RelB
cRel
Tnfa
e
visceral adipose tissue
20-ΔCq
*
0.0726
0.3187
RelB
cRel
Tnfa
f
cold exposure
8 °C
1 week
g
hypo
CD25+ Foxp3+ (% of CD4+)
*
room temperature
cold exposure
h
rest brain
CD25+ Foxp3+ (% of CD4+)
room temperature
cold exposure
i
CD80+ (% of macrophages)
*
room temperature
cold exposure
j
CD86+ (% of macrophages)
*
room temperature
cold exposure
k
MHCII+ (% of macrophages)
room temperature
cold exposure
l
CD80+ (% of microglia)
room temperature
cold exposure
m
CD86+ (% of microglia)
*
room temperature
cold exposure
n
MHCII+ (% of microglia)
room temperature
cold exposure

Supplementary Figure 8: Treg modulation by anti-IL2/IL2 antibody complexes or cold exposure. Related to Figure 7:

(**a**) Scheme of the *in vivo* Treg expansion experiment using anti-IL2/IL2 antibody complexes.

(**b-e**) Gene expression analyses of (b) hypothalami, (c) brains, (d) subcutaneous adipose tissue and (e) visceral adipose tissue of the mice from (a). Gene expression was normalized to *Histone H3*. N=3 biological replicates per group. Mean±SEM. Two-tailed student's unpaired *t*-test. Hypo: *p*(*RelB*)=0.3270; *p*(*cRel*)=0.2005; *p*(*Ikkb*)=0.1924. Brain: *p*(*Ccl5*)=0.1707; *p*(*Relb*)=0.5232; *p*(*cRel*)=0.2935; *p*(*Ikkb*)=0.4337. Subcutaneous adipose tissue: *p*(*Relb*)=0.1446; *p*(*cRel*)=0.0155; *p*(*Tnfa*)=0.0309. visceral adipose tissue: *p*(*Relb*)=0.0726; *p*(*cRel*)=0.0129; *p*(*Tnfa*)=0.3187.

(**f**) Scheme of the *in vivo* cold exposure experiments. Mice were subjected to 8°C ambient temperature for one week.

(**g-h**) $CD25^{hi}$ $Foxp3^{+}$ Treg frequencies in (g) hypothalamus (*p*=0.0435) and (h) rest brain (*p*=0.3400) after *in vivo* cold exposure. Two-tailed student's unpaired *t*-test. N=8 or 4 biological replicates. Depicted are box-and-whisker plots (min to max with all data points).

(**i-k**) Flow cytometric analysis of the expression of co-stimulatory molecules (i: CD80, j: CD86) or MHCII (k) on $CD45^{hi}CD11b^{+}$ macrophages after *in vivo* cold exposure. N=6 biological replicates. Depicted are box-and-whisker plots (min to max with all data points). *p*(CD80)=0.0460, *p*(CD86)=0.0168, *p*(MHCII)=0.9319.

(**l-n**) Flow cytometric analysis of the expression of co-stimulatory molecules (l: CD80, m: CD86) or MHCII (n) on $CD45^{int}CD11b^{+}$ microglia after *in vivo* cold exposure. N=6 biological replicates. Depicted are box-and-whisker plots (min to max with all data points). *p*(CD80)=0.1775, *p*(CD86)=0.0292, *p*(MHCII)=0.4065.

Source data are provided as a Source Data file. *=$p<0.05$.

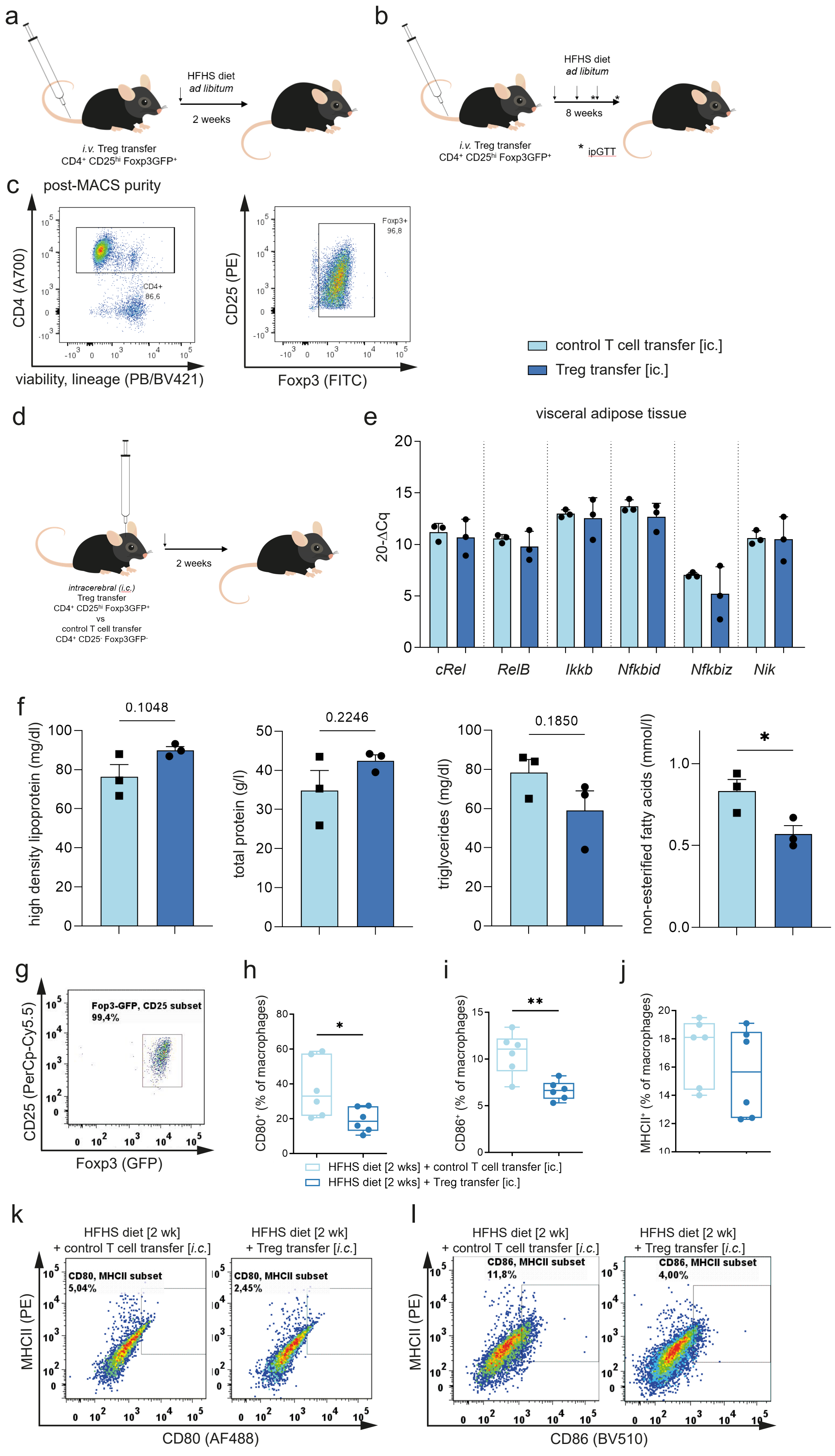
a
HFHS diet
ad libitum
2 weeks
i.v. Treg transfer
CD4⁺ CD25hi Foxp3GFP⁺
b
HFHS diet
ad libitum
8 weeks
i.v. Treg transfer
CD4⁺ CD25hi Foxp3GFP⁺
* ipGTT
c
post-MACS purity
CD4 (A700)
CD4+
86,6
viability, lineage (PB/BV421)
CD25 (PE)
Foxp3+
96,8
Foxp3 (FITC)
control T cell transfer [ic.]
Treg transfer [ic.]
d
2 weeks
intracerebral (i.c.)
Treg transfer
CD4⁺ CD25hi Foxp3GFP⁺
vs
control T cell transfer
CD4⁺ CD25⁻ Foxp3GFP⁻
e
visceral adipose tissue
20-ΔCq
cRel
RelB
Ikkb
Nfkbid
Nfkbiz
Nik
f
0.1048
high density lipoprotein (mg/dl)
0.2246
total protein (g/l)
0.1850
triglycerides (mg/dl)
*
non-esterified fatty acids (mmol/l)
g
Fop3-GFP, CD25 subset
99,4%
CD25 (PerCp-Cy5.5)
Foxp3 (GFP)
h
CD80⁺ (% of macrophages)
*
i
CD86⁺ (% of macrophages)
**
j
MHCII⁺ (% of macrophages)
HFHS diet [2 wks] + control T cell transfer [ic.]
HFHS diet [2 wks] + Treg transfer [ic.]
k
HFHS diet [2 wk]
+ control T cell transfer [i.c.]
HFHS diet [2 wk]
+ Treg transfer [i.c.]
CD80, MHCII subset
5,04%
CD80, MHCII subset
2,45%
MHCII (PE)
CD80 (AF488)
l
HFHS diet [2 wk]
+ control T cell transfer [i.c.]
HFHS diet [2 wk]
+ Treg transfer [i.c.]
CD86, MHCII subset
11,8%
CD86, MHCII subset
4,00%
MHCII (PE)
CD86 (BV510)

Supplementary Figure 9: Treg transfer models as a gain-of-function model to improve metabolic health. Related to Figure 8 and Figure 9

(**a-b**) Scheme of *i.v.* Treg transfer experiments.

(**c**) Post-MACS sort purity for the enrichment of $CD4^{+}CD25^{hi}$ Tregs analyzed by intracellular Foxp3 staining post-transfer of the *i.v.* Treg transfer cohort.

(**d**) Scheme of *i.c*. Treg vs control T cell transfer experiments.

(**e**) Gene expression analyses of visceral adipose tissue of the mice from (d). Gene expression was normalized to *Histone H3*. Mean±SEM. N=3 biological replicates per group. Two-tailed student's unpaired *t*-test. *P*(*cRel*)=0.6686; *p*(*Relb*)=0.4268; *p*(*Ikkb*)=0.7329; *p*(*Nfkbid*)=0.2890; *p*(*Nfkbiz*)=0.2971; *p*(*Nik*)=0.9315.

(**f**) Plasma analyses of (d). Mean±SEM. N=3 biological replicates per group. Two-tailed student's unpaired *t*-test. *P*(HDL)=0.1048; *p*(total protein)=0.2246; *p*(triglycerides)=0.1850; *p*(NEFAs)=0.0392.

(**g**) Representative plot of the FACS post sort-purity of dump-$CD4^{+}CD25^{hi}Foxp3GFP^{+}$ Tregs used for *i.c.* transfer experiments.

(**h-j**) Flow cytometric analysis of (h) CD80, (i) CD86 and (j) MHCII expression on $CD45^{high}CD11b^{+}$ macrophages after *i.c.* Treg or control T cell transfer and exposure to 2 wk HFHS diet. N=6 biological replicates. Depicted are box-and-whisker plots (min to max with all data points). Two-tailed student's unpaired *t*-test with *p*(CD80)=0.0363; *p*(CD86)=0.0027; *p*(MHCII)=0.3338.

(**k-l**) Analysis of costimulatory molecule co-expression on hypothalamic $CD45^{int}CD11b^{+}$ microglia after *i.c.* Treg or control T cell transfer and exposure to 2 wk HFHS diet.

Source data are provided as a Source Data file. *=*p*<0.05.

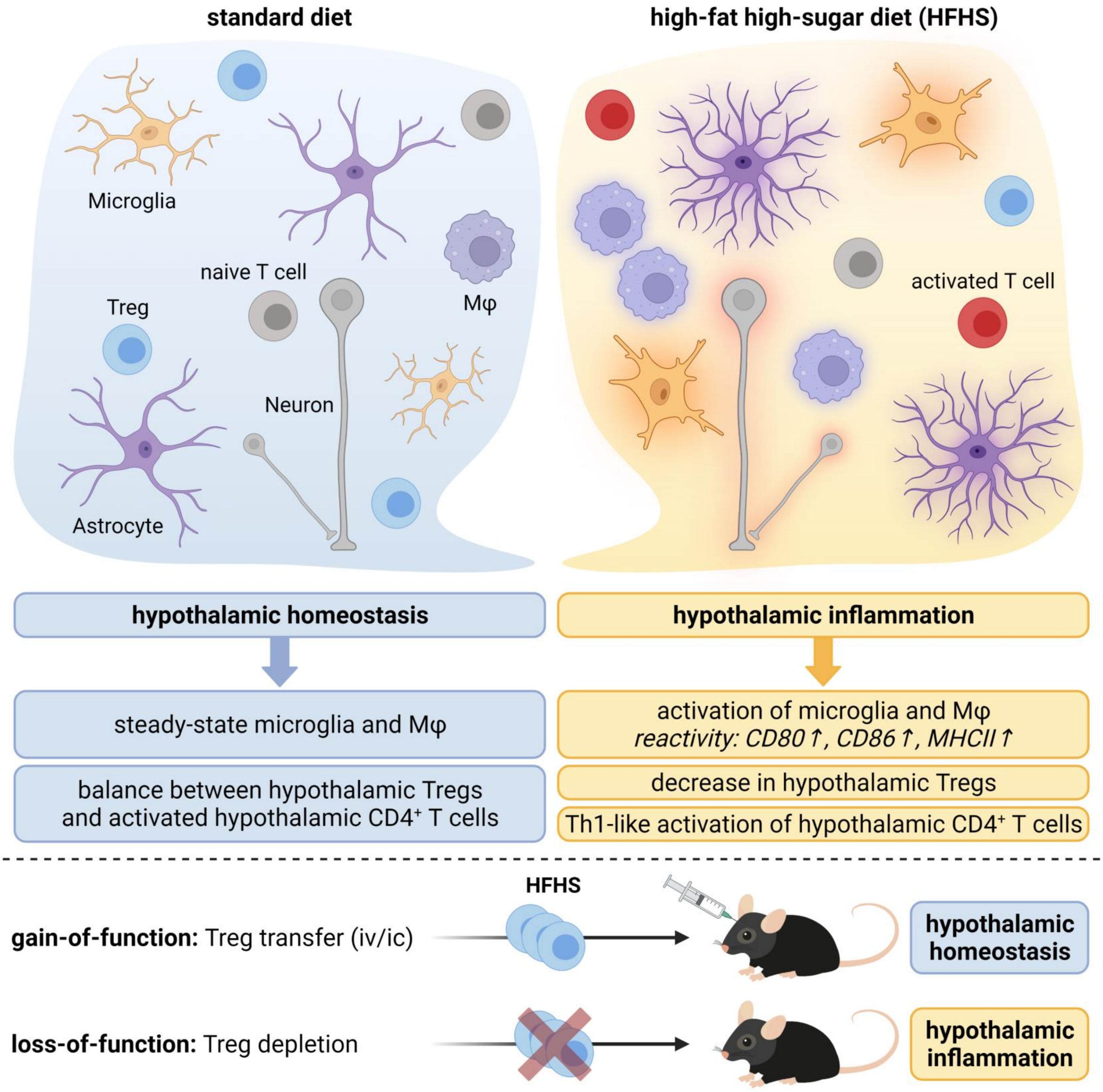
standard diet
high-fat high-sugar diet (HFHS)
Microglia
naive T cell
Treg
Mφ
Neuron
Astrocyte
activated T cell
hypothalamic homeostasis
hypothalamic inflammation
steady-state microglia and Mφ
balance between hypothalamic Tregs and activated hypothalamic CD4+ T cells
activation of microglia and Mφ
reactivity: CD80↑, CD86↑, MHCII↑
decrease in hypothalamic Tregs
Th1-like activation of hypothalamic CD4+ T cells
HFHS
gain-of-function: Treg transfer (iv/ic)
hypothalamic homeostasis
loss-of-function: Treg depletion
hypothalamic inflammation

Supplementary Figure 10: Regulatory T-cells in the hypothalamus control immune activation and improve metabolic impairments upon high-calorie environments.
A visual summary of the key findings from this study, highlighting that a hypercaloric challenge leads to microglia and macrophage reactivity, a significant reduction in hypothalamic Tregs, and a Th1-like activation of conventional CD4+ T cells in the hypothalamus. Gain-of-function experiments, including i.v. and i.c. Treg transfers, can restore hypothalamic immune activation following exposure to an HFHS diet, while loss-of-function experiments involving Treg depletion exacerbate hypothalamic inflammation. Created in BioRender. Scherm, M. (2025) https://BioRender.com/y95o387.

Supplementary Table 1

antibody	manufacturer	clone, cat#, RRID
CD4 Biotin	BioLegend	Clone: GK1.5; Cat# 553728; RRID:AB_395012
CD8a Pacific Blue	BioLegend	Clone: 53-6.7; Cat# 100725; RRID:AB_493425
CD11b Pacific Blue	BioLegend	Clone: M1/70; Cat# 101224; RRID:AB_755986
CD11c Brilliant Violet 421	BioLegend	Clone: N418; Cat# 117330; RRID:AB_11219593
B220 Pacific Blue	BioLegend	Clone: RA3-6B2; Cat# 103227; RRID:AB_492876
F4/80 Pacific Blue	BioLegend	Clone: BM8; Cat# 123124; RRID:AB_893475
CD25 PerCP-Cy5.5	BioLegend	Clone: PC61; Cat# 102030; RRID:AB_893288
CD44 PE	BioLegend	Clone: IM7; Cat# 103008; RRID:AB_312959
Ki67 APC	BioLegend	Clone: 16A8; Cat# 652406; RRID:AB_2561930
Ki67 Brilliant Violet 605	BioLegend	Clone: 16A8; Cat# 652413; RRID:AB_2562664
CD4 Alexa Fluor 700	eBioscience	Clone: RM4-5; Cat# 56-0042-82; RRID:AB_494000
CD62L APC	eBioscience	Clone: MEL-14; Cat# 17-0621-82; RRID:AB_469410
Foxp3 FITC	eBioscience	Clone: FJK-16s; Cat# 11-5773-82; RRID:AB_465243
CD14 V450	BD Biosciences	Clone: rmC5-3; Cat# 560639; RRID:AB_1727429
CD4 Pacific Blue	BioLegend	Clone: GK1.5; Cat# 100428 RRID:AB_493647
CD11b PerCp-Cy5.5	BioLegend	Clone: M1/70; Cat# 101228 RRID:AB_893232
CD45 APC	BioLegend	Clone: 30-F11; Cat# 103112 RRID:AB_312977
CD45 Alex Fluor 700	BioLegend	Clone: 30-F11; Cat# 103128 RRID:AB_493715
CD45 PE-Cy7	BioLegend	Clone: 30-F11; Cat# 103113 RRID:AB_312978

CD45.1 APC-Cy7	BioLegend	Clone; A20; Cat# 110716; RRID: AB_313505
CD86 Brilliant Violet 510	BioLegend	Clone: GL-1; Cat# 105039 RRID:AB_2562370
CD86 APC-Cy7	BioLegend	Clone: GL-1; Cat# 105029 RRID:AB_2074993
CD80 Alexa Fluor 488	BioLegend	Clone: 16-10A1; Cat# 104715 RRID:AB_492823
MHCII I-A PE	eBioscience	Clone: NIMR-4; Cat# 12-5322-81 RRID:AB_465930
NeuN-	Sigma Aldrich	Clone: A60; Cat# MAB377X; RRID:AB_2149209
Goat anti-GFP	Acris antibodies	Polyclonal; Cat# R1091P RRID:AB_1002036
Rabbit anti-GFAP	Dako	Cat# Z0334; RRID:AB_10013382
Rabbit anti-Iba1	Synaptic Systems	Polyclonal; Cat# 234 003 RRID:AB_10641962
Armenian hamster anti-CD3	BioLegend	Clone: 145-2C11; Cat# 100301 RRID:AB_312666
Rat anti-Foxp3	eBioscience	Clone: FJK-16s; Cat# 14-5773-82 RRID:AB_467576
Rat anti-CD4	BD	Clone: GK1.5; Cat# 553727 RRID:AB_395011
Rabbit anti-Collagen IV	Millipore	Clone: AB756P; Cat# AB756P RRID:AB_2276457
Anti-mouse CD25 (mCD25)	BioXCell	Clone: PC-61.5.3; Cat# BE0012; RRID:AB_1107619
CellTrace Violet	Invitrogen	Cat#C34557
Fc-Block	BD Pharmingen	Clone: 2.4G2; Cat# 553142; RRID:AB_394657
Donkey anti-goat Alexa Fluor 488	Life Technologies	Cat# A11055; RRID:AB_2534102
Donkey anti-rabbit Alexa Fluor 568	Life Technologies	Cat# A10042; RRID:AB_2534017
Goat anti-hamster Alexa Fluor 488	Jackson ImmunoResearch Labs	Cat# 127-545-160; RRID:AB_2338997
Biotinylated rabbit anti-goat	Vector	Cat# BA-5000; RRID:AB_2336126
Biotinylated goat anti-rat	Jackson ImmunoResearch Labs	Cat# 112-065-175; RRID:AB_2338180
Goat anti-rabbit Alexa Fluor 488	Jackson ImmunoResearch Labs	Cat# 111-545-144; RRID:AB_2338052

Supplementary Table 2

Mouse line	Source	RRID
CD90.1 Balb/c; genotype: CBy.PL(B6)-*Thy1*^{a}/ScrJ	Jackson Laboratory	RRID:IMSR_JAX:005443
CD90.2 Balb/c; genotype: Balb/cByJ	Jackson Laboratory	RRID:IMSR_JAX:001026
Foxp3 GFP Balbc; genotype: C.Cg-Foxp3^{tm2Tch}/J	Jackson Laboratory	RRID:IMSR_JAX:006769
Foxp3 GFP Bl6; genotype: B6.Cg-Foxp3^{tm2Tch}/J	Jackson Laboratory	RRID:IMSR_JAX:006772
CD45.1 Bl6 "wt"; genotype: B6.SJL-*Ptprc*^{a}*Pepc*^{b}/BoyJ	Jackson Laboratory	RRID:IMSR_JAX:002014
*ob/*ob mice; genotype: B6.Cg-*Lep*^{ob}/J	Jackson Laboratory	RRID:IMSR_JAX:000632
Foxp3-DTR; genotype: C57BL/6-Tg(Foxp3-DTR/EGFP)23.2Spar/Mmjax	Tobias Bopp, Johannes Gutenberg University Mainz, Germany	RRID:MMRRC_032050-JAX
Wildtype C57Bl/6J	Jackson Laboratory	RRID:IMSR_JAX:000664
Wildtype Balb/cByJ	Jackson Laboratory	RRID:IMSR_JAX:001026

Supplementary Table 3

gene	forward primer	reverse primer	source
Relb	gcc ttg ggt tcc agt gac	tgt att cgt cga tga ttt cca a	Vigo Heissmeyer; LMU Munich
cRel	TTTCCTTCCTGATGAACATGG	CACGGCAGATCCTTAATTCT	Vigo Heissmeyer; LMU Munich
Ikkb	ccg gaa agt gtc agc tgt atc	cct cag ctg gaa gaa gga ga	Vigo Heissmeyer; LMU Munich
Nik	tcc aca gaa tga agg aca agc	tac ccg aaa cac ctc gag tc	Vigo Heissmeyer; LMU Munich
Nfkbid	ttt cta ccc tcc gtc aga cc	tac agc cgg gta tcc aga ga	Vigo Heissmeyer; LMU Munich
Nfkbiz	gag tcc cgt ccc aga ggt	ttc acg cga aca cct tga	Vigo Heissmeyer; LMU Munich
Ccl5	TGCAGTCGTGTTTGTCACTC	ATGCCCATTTTCCCAGGACC	Self-designed
Tnfa	ATGAGAAGTTCCCAAATGGC	CTCCACTTGGTGGTTTGCTA	Self-designed
